# Ginsenoside Rg5 allosterically interacts with P2RY_12_ and ameliorates deep venous thrombosis by counteracting neutrophil NETosis and inflammatory response

**DOI:** 10.3389/fimmu.2022.918476

**Published:** 2022-08-12

**Authors:** Ziyu Chen, Gaorui Wang, Xueqing Xie, Heng Liu, Jun Liao, Hailian Shi, Min Chen, Shusheng Lai, Zhengtao Wang, Xiaojun Wu

**Affiliations:** ^1^ Shanghai Key Laboratory of Compound Chinese Medicines, The Ministry of Education (MOE) Key Laboratory for Standardization of Chinese Medicines, The State Administration of TCM (SATCM) Key Laboratory for New Resources and Quality Evaluation of Chinese Medicine, Institute of Chinese Materia Medica, Shanghai University of Traditional Chinese Medicine, Shanghai, China; ^2^ School of Life Science and Technology, Shanghai Tech University, Shanghai, China; ^3^ Guangxi Key Laboratory of Comprehensive Utilization Technology of Pseudo-Ginseng, Wuzhou, China

**Keywords:** deep venous thrombosis, ginsenoside Rg5, P2RY_12_, neutrophil extracellular traps, inflammation

## Abstract

**Background:**

Deep venous thrombosis (DVT) highly occurs in patients with severe COVID-19 and probably accounted for their high mortality. DVT formation is a time-dependent inflammatory process in which NETosis plays an important role. However, whether ginsenoside Rg5 from species of *Panax* genus could alleviate DVT and its underlying mechanism has not been elucidated.

**Methods:**

The interaction between Rg5 and P2RY_12_ was studied by molecular docking, molecular dynamics, surface plasmon resonance (SPR), and molecular biology assays. The preventive effect of Rg5 on DVT was evaluated in inferior vena cava stasis–induced mice, and immunocytochemistry, Western blot, and calcium flux assay were performed in neutrophils from bone marrow to explore the mechanism of Rg5 in NETosis *via* P2RY_12_.

**Results:**

Rg5 allosterically interacted with P2RY_12_, formed stable complex, and antagonized its activity *via* residue E188 and R265. Rg5 ameliorated the formation of thrombus in DVT mice; accompanied by decreased release of Interleukin (IL)-6, IL-1β, and tumor necrosis factor-α in plasma; and suppressed neutrophil infiltration and neutrophil extracellular trap (NET) release. In lipopolysaccharide- and platelet-activating factor–induced neutrophils, Rg5 reduced inflammatory responses *via* inhibiting the activation of ERK/NF-κB signaling pathway while decreasing cellular Ca^2+^ concentration, thus reducing the activity and expression of peptidyl arginine deiminase 4 to prevent NETosis. The inhibitory effect on neutrophil activity was dependent on P2RY_12_.

**Conclusions:**

Rg5 could attenuate experimental DVT by counteracting NETosis and inflammatory response in neutrophils *via* P2RY_12_, which may pave the road for its clinical application in the prevention of DVT-related disorders.

## Graphical Abstract

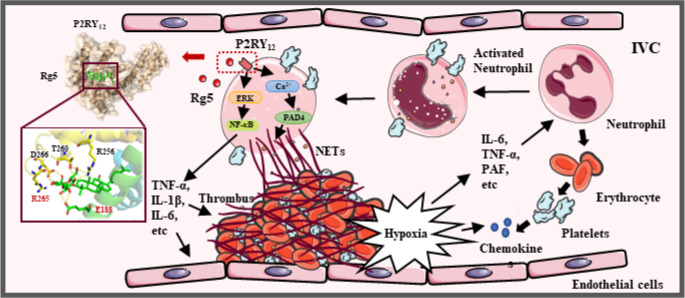

## Introduction

Deep venous thrombosis (DVT) belongs to venous thromboembolic disorder and is the leading cause of pulmonary embolism, which eventually contributes to heart failure and even sudden death (1). DVT is generally characterized by abnormal coagulation of blood in deep veins of lower leg and thigh; however, it may occur in upper limb deep veins, visceral veins, and vena cava (2). DVT highly occurs in long-term sedentary people and post-surgery patients (3). Recently, clinical studies have found an association between high mortality in patients with COVID-19 and DVT (4). The clinical diagnosis of DVT mainly depends on clinical risk score, serum D-dimer, and color Doppler ultrasound. Anticoagulants such as Aspirin, antiplatelet drugs, Rivaroxaban, and low molecular–weight heparin are common clinical treatment for the prevention of DVT (5). Unfortunately, all of these treatments carry a remarkable risk of bleeding (6). Therefore, it is still urgent to develop effective antithrombotic drugs with less side effects to slow down the occurrence of DVT.

Neutrophils play an import role in thrombosis. Together with platelets, neutrophils are the first cells mobilized to the injury/infection sites to limit the dissemination of microbial infection by promoting blood coagulation. However, dysregulation or excessive stimulation may contribute to thrombotic processes (7). Neutrophils release neutrophil extracellular traps (NETs) upon excessive stimulation, a process called NETosis, which promotes thrombosis by forming a scaffold for adhesion of platelets, erythrocytes, and platelet adhesion molecules (8). NETs are extracellular, web-like structures mainly composed of decondensed chromatin and granule proteins such as histones, neutrophil elastase, myeloperoxidase (MPO), calprotectin, cathelicidins, defensins, and actin (9). When activated by Ca^2+^, peptidyl arginine deiminase 4 (PAD4) catalyzes the conversion of histone arginine to citrulline, resulting in the decondensation of heterochromatin and prompting NET formation. Preventing NETosis can decrease thrombogenicity, which may be beneficial for thrombosis prevention and become a promising therapy target for DVT (10).

P2RY_12_, as an adenosine diphosphate (ADP) receptor on the platelet surface, is a key player in platelet activation and a vital target of antithrombotic drugs such as Clopidogrel, Praugrel, and Ticagrelor (11). Upon activation by ADP, P2RY_12_ is coupled to the G_i2_ protein, and its activation inhibits adenylate cyclase activity, thereby reducing the intracellular cyclic adenosine monophosphate (cAMP) level. In addition, G_i2_ signaling gives rise to the activation of Phosphatidylinositol 3-kinase/protein kinase B (PI3K/AKT) pathway, which leads to a significant increase of granule release and platelet aggregation (12, 13), suggesting that dense granule release, procoagulant activity, and thrombosis are dependent on P2RY_12_ activation (14, 15). Interestingly, P2RY_12_ is expressed not only on the surface of platelets but also on several immune cells, including eosinophils, monocytes, macrophages, lymphocytes dendritic cells, and mast cells, which are involved in the inflammatory process of many diseases (16). Studies have shown that the P2RY_12_ plays an important role in early DVT formation (17), and P2RY_12_ inhibitor Clopidogrel, Ticagrelor, or Prasugrel can restrain platelet-leukocyte interaction (18–20). Although a study has demonstrated the reduction of platelet-leukocyte aggregation and NETs by Ticagrelor in patients with pneumonia (21), the direct relationship between P2RY_12_ and NETosis has not been elucidated yet.

Species of *Panax* genus, including *Panax ginseng* C.A. Meyer, *Panax notoginseng* (Burkill) F.H. Chen, and *Panax quinquefolius* L., are precious Chinese herbal medicines with beneficial effects in reinforcing immunity and reducing fatigue. Ginsenoside Rg5 (**Figure 2A**) is one of the natural saponins in *P. ginseng* and *P. notoginseng*, which has multiple pharmacological activities, such as anti-cancer, anti-inflammation, anti-diabetes, anti-obesity, neuroprotection, and cardioprotection (22). However, whether Rg5 could benefit DVT therapy has not been elucidated yet. In the present study, we firstly identified that Rg5 might bind to P2RY_12_ by molecular docking, which was confirmed by molecular dynamics (MD) simulation, surface plasmon resonance (SPR), and site mutation analysis. Furthermore, in DVT model mice, we found that Rg5 could attenuate thrombosis, which might be exerted by inhibiting NETosis through preventing NET release and inflammatory response by antagonizing P2RY_12_. These findings may pave the road for the clinical application of Rg5 in the prevention of DVT.

## Materials and methods

### Chemicals

Ginsenoside Rg5 (Cat# BP1651, purity > 95%) was purchased from Biopurify Phytochemicals, Ltd. (Chengdu, China). Rivaroxaban (Cat# MB1878, purity > 98%) was obtained from Meilun Biotechnology Co., Ltd. (Dalian, China). Platelet-activating factor (PAF) (Cat# GC14535) was provided by GlpBio Technology, Inc. (CA, USA). Lipopolysaccharide (LPS) from *Escherichia coli* 0111:B4 was obtained from Sigma-Aldrich Company (MO, USA). 2-Methylthioadenosine diphosphate (2MesADP) trisodium salt was purchased from Tocris Bioscience (MN, USA). n-Dodecyl-β-D-Maltopyranoside (DDM) (Cat# D310) was bought from Anatrace (OH, USA).

### Antibodies

Antibodies against AKT (Cat# T55561F), phospho-AKT-Ser473 (Cat# T40067F), phospho-ERK1 (T202/Y204) + ERK2 (T185) (Cat# T40072F), p44/42 MAPK (ERK1/2) (Cat# T40071F), and GAPDH (Cat# M20006F) were purchased from Abmart (Shanghai, China). Nuclear factor-kappa B (NF-kB) (Cat# 8242S), p-NF-κB-Ser536 (Cat# 3033L), and β-actin (Cat# 12413) antibodies were obtained from Cell Signaling Technology (C.S.T.) Co. (MA, USA). Anti-PAD4 antibody (Cat# ab214810) and anti-Histone H3 (citrulline R2 + R8 + R17) antibody (Cat# ab5103) were purchased from Abcam (Cambridge, England). Anti-MPO antibody (Cat# GB11224) was purchased from Servicebio (Wuhan, China).

### Animals

Wide-type C57BL/6 male mice (25 ± 2 g) were provided by Shanghai Sippe-Bk Lab Animal Co., Ltd. and were adapted for 1 week before use. P2RY_12_-knockout (KO) mice were kindly provided by Dr. Jun-ling Liu from Ruijin Hospital, Shanghai Jiaotong University School of Medicine (Shanghai, China), and genotyped as described previously (23). All mice received humane care and were kept in a standard environment with a 12/12-h cyclic lighting schedule (lights on at 07:00 am) in the Laboratory Animal Center of Shanghai University of Traditional Chinese Medicine (SHUTCM, Shanghai, China). The temperature and humidity were maintained at 25 ± 2°C and 45 ± 5%, respectively.

### Bone marrow neutrophils preparation

BMNs from the femur and tibia of wild type (WT) or P2RY_12_-KO mice were prepared according to the instruction of the Mouse Bone Marrow Neutrophil Extraction Kit (Solarbio, Beijing, China).

### DNA content detection

BMNs were seeded in 24-well plate at 2 × 10^6^/ml per well and treated with PBS or 6.25, 12.5, and 18.75 μM Rg5 for 2 h followed by stimulation of LPS (20 μg/ml) or 50 μM PAF for 30 min. Then, the cells were collected for Western blot analysis, and cell-free supernatant was collected for further analysis. DNA content in supernatant was measured by using Quant-iT PicoGreen dsDNA Reagent and Kits (Invitrogen, CA, USA). The concentration of DNA released by neutrophils treated with 0.3% Triton was set as 100%, and the relative proportion of cell-free DNA (cfDNA) concentration in each sample was calculated by comparing with the former.

### ELISA assay

Serum or medium concentrations of D-dimer, IL-6, IL-1β, tumor necrosis factor-α (TNF-α) and citrullinated histones 3 (CitH3) were measured by using respective ELISA kits (Lengton Bioscience, Shanghai, China). The absorbance was detected at 450 or 570 nm on a microplate reader (FlexStation 3, Molecular Devices, CA, USA).

### Immunocytochemistry

To examine the effect of Rg5 on the formation of NETs, the BMNs were seeded in 24-well plates with coverslips pre-coated with poly-D-lysine. After treatment of 18.75 μM Rg5 for 2 h followed by stimulation of LPS (5 μg/ml) or 20 μM PAF for 3 h, the cells were fixated by 4% Paraformaldehyde (PFA) for 10 min. Consequently, the cells were blocked with 5% donkey serum for 1 h and incubated overnight with antibody against CitH3 (1:1,000) at 4°C, followed by Alexa 488–conjugated secondary antibody (1:800). Finally, the coverslips were mounted on slides with mounting medium containing Diamidinyl phenyl indole (DAPI) in the dark. The microscopy images were captured by Olympus slide scanner (VS120, Japan) and analyzed by ImageJ (version 1.46r).

### Molecular docking and consensus analysis

The P2RY_12_–antagonist complex structure (Protein Data Bank Code: 4NTJ) was obtained to predict the potential binding mode of Rg5 with P2RY_12_. As described previously (24), AutoDock Vina (version 1.1.2), Maestro (version 11.4, Schrödinger, LLC, New York, NY, 2021), and molecular operating environment (MOE, Chemical Computing Group, version 2019.0101) softwares were used to calculate the binding capability of Rg5 and other compounds. The exponential consensus ranking (ECR) analysis (25) was used to assign a rank to each ligand based on the molecular docking score provided by different docking programs to combine the results of multiple docking programs.

### P2RY_12_ protein expression and purification

P2RY_12_ protein were expressed and purified as described previously (26) and concentrated to approximately 1 mg/ml for further usage.

### Surface plasmon resonance

The binding affinity between Rg5 and P2RY_12_ was measured using Biacore T200 (GE healthcare, MA, USA). P2RY_12_ protein was immobilized on the chip in the presence of 1× HEPES Buffered Saline-EDTA P20 (HBS-EP) with 0.03% DDM buffer (pH 7.4) by using the His Capture Kit (GE healthcare, MA, USA). Various concentrations of Rg5 (0.098, 0.195, 0.391, 0.781, 0.562, and 3.125 μM) or Ticagrelor (0.031, 0.062, 0.125, 0.25, 0.5, and 1 μM) dissolved in 5% DMSO were passed at 30 μl/min for 120 s over the P2RY_12_ protein, followed by a dissociation step of 120 s. The Equilibrium dissociation constant (KD) value was obtained by fitting the data to a steady state affinity model using Biacore T200 Evaluation Software (version 3.0).

### Molecular dynamics simulations

The stable MD trajectory of the P2RY_12_–Rg5, P2RY_12_–Ticagrelor, or P2RY_12_–Aspirin complex was estimated by the Molecular mechanics/poisson-boltzmann surface area (MM/PBSA) technique implemented in AMBER14 as described previously (27). The MM/PBSA method combined molecular mechanics and continuous solvent model was used to predict the binding free energy of P2RY_12_ protein and ligands.

### P2RY_12_ signaling transduction detection

CHO-P2RY_12_ cells overexpressing P2RY_12_ were cultured in Dulbecco's modified eagle medium (DMEM) high-glucose medium containing 10% fetal bovine serum at 37°C. To examine the inhibitory effect of Rg5 against P2RY_12_ signaling, CHO-P2RY_12_ cells were seeded at a density of 1 × 10^5^ cells per well in six-well plates for 24 h and serum-starved for 12 h. Then, the cells were treated with Rg5 (3.125, 6.25, 12.5, 25, and 50 μM) for 2 h followed by stimulation of 2MesADP (100 nM) for 5 min. Afterward, the cells were collected for further Western blot analysis.

### P2RY_12_ site mutation

The coding sequence of P2RY12 (Homo sapiens, Gene ID: 64805) was cloned into PCMV6 vector and site-directed mutated by using the Mut Express II Fast Mutagenesis Kit (Vazyme, Nanjing, China). The primers (Generay Biotech, Shanghai, China) were listed as follows: P2RY12_E188A mutation (forward, 5′- GTCTGGCATGCGATAGTAAATTACATCTGTC-3′; reverse, 5′- GTAATTTACTATCGCATGCCAGACTAGACCG-3′), P2RY12_R265A mutation (forward, 5′- CAAACCGCTGATGTCTTTGACTGCACTGCTGAA-3′; reverse, 5′- GACATCAGCGGTTTGGCTCAGGGTGTAAGGAATT-3′), P2RY12_D266A mutation (forward, 5′- ACCCGGGCGGTCTTTGACTGCACTGCTGAAAATA -3′; reverse, 5′- AAAGACCGCCCGGGTTTGGCTCAGGGTGTAA -3′), and P2RY12_R265A and D266A mutation (forward, 5′- CTGAGCCAAACCGCTGCGGTCTTTGACTGC -3′; reverse, 5′- GTGCAGTCAAAGACCGCAGCGGTTTGGCTC -3′). Plasmid was extracted according to the steps in the EndoFree Midi Kit (Cwbiotech, Taizhou, China) and transfected into CHO cells for 12 h by Tecfect DNA transfection reagent (TEYE Co, Shanghai, China). CHO cells were cultured and treated under the same conditions as CHO-P2RY_12_ cells, which were collected for further Western blot analysis.

### Western blot analysis

The methods of protein samples preparation and Western blot analysis were described previously (28). After incubation in primary antibodies (1:1,000) and secondary antibody (1:5,000), the protein bands were visualized by using the ECL Enhanced Kit (ABclonal Technology, Wuhan, China). The photographs were taken and analyzed by using Tanon 5200 Multi (Shanghai, China).

### Inferior vena cava stasis-induced DVT

DVT model was established as described previously (29). In brief, the IVC caudal to the left renal vein was ligated for 12 h to achieve stasis induction of thrombosis. None of the mice showed any bleeding during surgery.

### Drug administration

A total of 48 mice were randomly divided into six groups: (1) Sham group; (2) DVT group; (3) Rg5 of 1.25 mg/kg + DVT group; (4) Rg5 of 2.5 mg/kg + DVT group; (5) Rg5 of 5 mg/kg + DVT group; and (6) Rivaroxaban of 0.3 mg/kg + DVT group. Rg5 is dissolved in saline containing 2% ethanol for injection, and Rivaroxaban was dissolved in a special solution (Polyethylene glycol/Saline/Glycerin = 996 g/100 g/60g). Rg5 or Rivaroxaban was intravenously given at 15 min prior to thrombus induction. IVC of mice in sham group was separated without ligation. After the formation of DVT, arterial blood was collected immediately and mixed with 10% sodium citrate for anticoagulation followed by centrifugation at 3,000 rpm for 15 min to obtain the plasma. The IVC between the left renal vein and the iliac crest bifurcation was separated, whose length and wet weight were measured. Afterward, the IVC was fixed by 4% PFA for further hematoxylin and eosin (HE) staining and immunohistochemistry.

### Histopathology and immunohistochemistry

For HE staining, the 5-μm-thick sections were dewaxed and stained by HE as described previously (30). For immunohistochemistry, the sections were deparaffinzed and rehydrated, followed by antigen retrieval as described elsewhere (31). After deactivation of endogenous peroxidase with H_2_O_2_, they were blocked with 3% Bull serum albumin (BSA) and incubated with the anti-MPO antibody (1:2,000) and the secondary antibody (1:200). The immuno-reactive cells were visualized after Diaminobenzidine (DAB) chromogenic reaction. Finally, the images were captured by Olympus slide scanner (VS120, Japan) and analyzed by ImageJ (version 1.46r).

### Calcium flux assay

Calcium flux in BMNs was monitored by using the Screen Quest™ Calbryte-520 Probenecid-Free and Wash-Free Calcium Assay Kit (AAT Bioquest, CA, USA). In brief, BMNs were seeded at 2 × 10^6^ cells/ml in 96-well plate. After Rg5 (18.75 μM) treatment for 2 h, BMNs were loaded with Calbryte 520 AM dye for 45 min. Then, the cells were stimulated with PAF (20 μM) and monitored immediately on a fluorescence microplate reader (FlexStation 3, Molecular Devices, CA, USA) with excitation wavelength at 490 nm and emission wavelength at 525 nm at 37°C for 60 min.

### PAD4 activity assay

BMNs were stimulated with PAF (20 μM) for 3 h after pre-treated with Rg5 (18.75 μM) for 2 h. Then, the cells were collected and lysed to extract the proteins, which were incubated with 10 mM ethyl N-benzoyl-L-argininate hydrochloride (BAEE) at 37°C for 10 min. The ammonia content produced through the enzymatic hydrolysis of BAEE by PAD4 was measured according to the procedure described by the manufacturer (Blood Ammonia Content Detection Kit). Finally, the absorbance was measured at 630 nm on a microplate reader (FlexStation 3).

### Statistical analysis

The data were presented as mean ± SEM to describe the differences among multiple groups. Differences among groups were analyzed by one-way ANOVA with Dunnett’s analysis (n ≥ 4) and Kruskal–Wallis test (n = 3) using GraphPad Prism 5.0. The value of *P <* 0.05 was considered statistically significant.

## Results

### P2RY_12_ actively participated in NETosis

**Figure 1:**
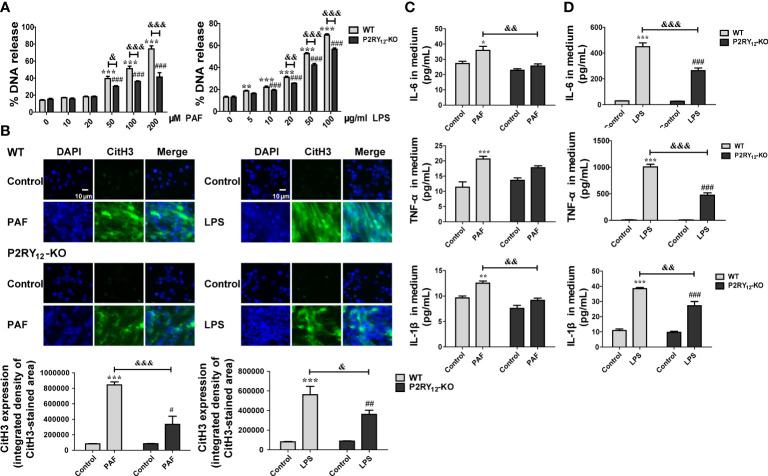
Effects of P2RY_12_ on PAF and LPS-induced NET release and inflammatory responses. **(A)** Different concentrations of PAF or LPS induced the release of cfDNA from WT or P2RY_12_-KO neutrophils (n = 4). **(B)** Immunofluorescent staining and statistical analysis of CitH3 and DAPI staining of WT or P2RY_12_-KO neutrophils induced by PAF and LPS, respectively (n = 4). **(C, D)** Release of IL-6, IL-1β, and TNF-α in medium of WT or P2RY_12_-KO neutrophils induced by PAF and LPS, respectively (n = 4). All the data are shown as the mean ± SEM. *P < 0.05, **P < 0.01, ***P < 0.001 vs. WT control; ^###^ *P <* 0.001 vs. P2RY_12_-KO Control; ^&^ *P <* 0.05, ^&&^ *P <* 0.01, ^&&&^ *P <* 0.001, WT vs. P2RY_12_-KO. #P < 0.05, ##P < 0.01.

Because whether P2RY_12_ is involved in NETosis has not been elucidated yet, we firstly investigated its role in NETosis. As shown in **Figure 1A**, PAF dose-dependently induced the production of cfDNA in both WT and P2RY_12_-KO neutrophils. However, when stimulated with the same dose of PAF, higher than 50 μM, P2RY_12_-KO neutrophils produced significantly less cfDNA than WT neutrophils (*P <* 0.05, *P <* 0.001). Similarly, when stimulated at the concentration higher than 20 μg/ml, LPS also induced much more release of cfDNA in WT neutrophils than that in P2RY_12_-KO neutrophils (*P <* 0.01 and *P <* 0.001). After PAF or LPS stimulation, much more NETs in reticular structure, which were mainly composed of DNA and CitH3 derived from chromatin depolymerization in the nucleus, were released from WT neutrophils than that from P2RY_12_-KO neutrophils (**Figure 1B**, *P <* 0.01). Moreover, PAF-induced remarkably increased production of inflammatory factors, such as IL-6, IL-1β, and TNF-α in WT neutrophils (**Figure 1C**, *P <* 0.05); by contrast, it induced slight increment of IL-6, IL-1β, and TNF-α in P2RY_12_-KO neutrophils. Similarly, LPS induced more release of inflammatory cytokines in WT neutrophils than that in P2RY_12_-KO neutrophils (**Figure 1D**). These results implicated that P2RY_12_ is critical for the NETosis and inflammatory response in neutrophils.

### Rg5 allosterically bound to P2RY_12_ and antagonized its activity

**Figure 2:**
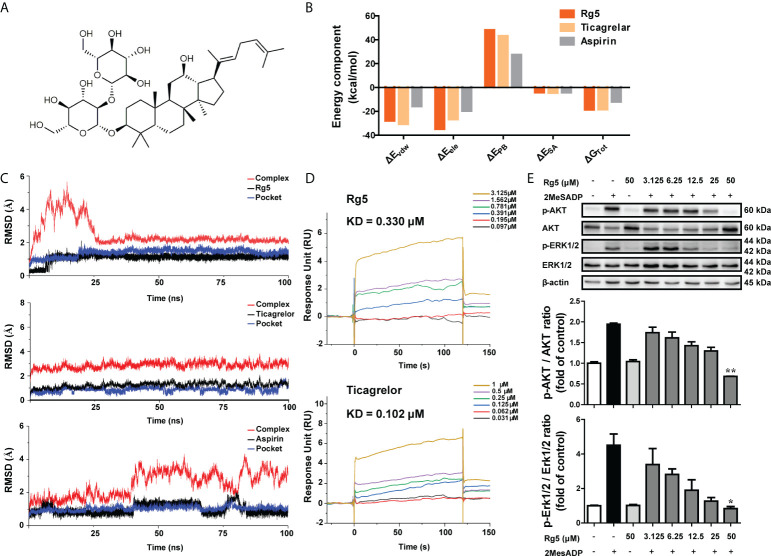
Interaction between Rg5 and P2RY_12_. **(A)** Chemical structure for Rg5. **(B)** Comparison of binding free energy between Rg5, Ticagrelor, and Aspirin binding to P2RY_12_. ΔE_vdw_, van der Waals energy; ΔE_ele_, electrostatic energy; ΔE_PB_, Poisson–Boltzmann energy; ΔE_SA_, nonpolar energy; ΔG_Tot_, total binding free energy. **(C)** Stability analysis of Rg5, Ticagrelor, and Aspirin binding to P2RY_12_. **(D)** SPR analyses of Rg5 and Ticagrelor binding to P2RY_12_. **(E)** Protein expression and phosphorylation levels of AKT (n = 3) and ERK (n = 3). All the data are shown as the mean ± SEM. *P < 0.05, **P < 0.01 vs. 2MesADP group.

As aforementioned, P2RY_12_ is important for neutrophil activation, particularly NETosis, we next carried out virtual screening by using ECR strategy, which was adopted to better describe the comprehensive binding affinity of the P2RY_12_–ligand complex predicted by Autodock Vina, Glide, and MOE softwares. We used ranking values rather than docking scores in subsequent docking analyses. According to the ranking results, the binding affinity between Rg5 and P2RY_12_ was similar to that between Ticagrelor and P2RY_12_ (**Table 1**). To further investigate the stability of Rg5 binding to P2RY_12_, MD analysis was performed. As shown in **Figure 2B**, the electrostatic interaction (ΔE_ele_) between Rg5 and P2RY_12_ was higher than that between Ticagrelor and P2RY_12_, and the total free energy (ΔG_Tot_) of Rg5 was also slightly higher than that of Ticagrelor. In addition, as shown in **Figure 2C**, compared with the stable state of Ticagrelor in P2RY_12_, although the complex of Rg5 and P2RY_12_ fluctuated before 25 ns, it quickly leveled off later; and Aspirin remained in a state of fluctuation in P2RY_12_. The Root mean square deviation (RMSD) trajectories of Rg5 and Pocket also fluctuated slightly at the beginning but became stable later on. To confirm the direct interaction, we conducted SPR assay. As shown in **Figure 2D**, Rg5 dose-dependently bound to P2RY_12_ with a KD of 0.33 μM, which was similar to that of Ticagrelor (0.102 μM). These results suggested that Rg5 could bind to P2RY_12_ steadily and affect the activity of the latter. On this premise, we used CHO-P2Y_12_-OE cells to explore whether the binding of Rg5 to P2RY_12_ could interfere the downstream signaling of the latter. As demonstrated in **Figure 2E**, Rg5 itself had no effect on P2RY_12_ signaling, but it inhibited 2MesADP-induced phosphorylation of AKT and ERK in a dose-dependent manner, suggesting the antagonic effect of Rg5 on P2RY_12_.

**Table 1:** Ranking results of Rg5, Ticagrelor, and Aspirin binding to P2RY_12_.

Receptor	Ligand	Rank
P2RY_12_	Rg5	0.684
	Ticagrelor	0.701
	Aspirin	0.525

**Figure 3:**
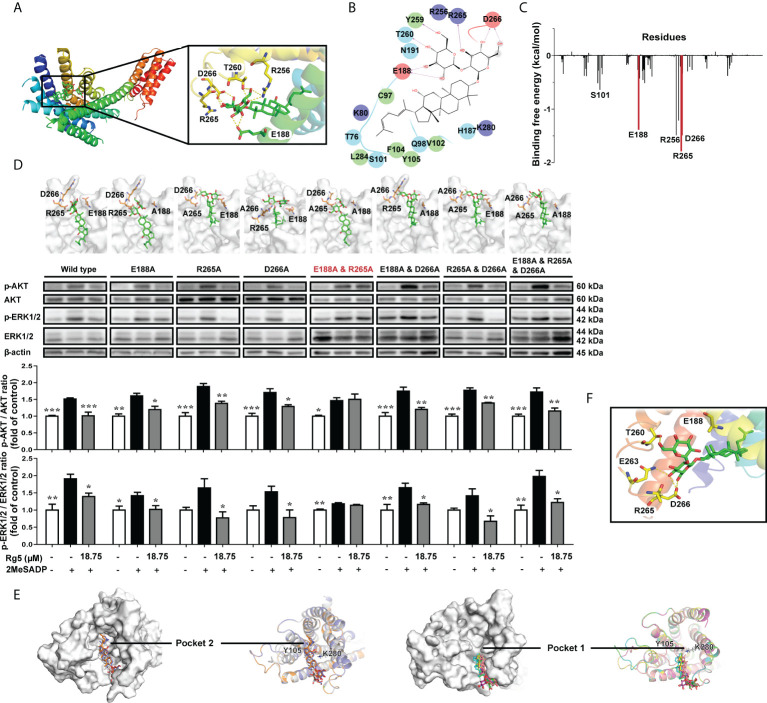
Interaction sites of Rg5 with P2RY_12_. **(A)** P2RY_12_ and Rg5 complex structure, and binding pocket with Rg5. **(B)** Two-dimensional interaction map of interactions between Rg5 and P2RY_12_. **(C)** Energy decomposition of amino acid residues in P2RY_12_ and Rg5 based on MM/PBSA free energy calculation. **(D)** Structures of P2RY_12_ mutation variants with Rg5 bound were shown in the upper panel, and Western blot analysis in the lower panel displayed the protein expression and phosphorylation levels of AKT (n = 4) and ERK (n = 4). All the data are shown as the mean ± SEM. *P < 0.05, **P < 0.01, ***P < 0.001 vs. 2MesADP group. **(E)** Hypothetical binding modes of Rg5 to P2RY_12_ mutation variants obtained by molecular docking. The binding conformation of Rg5 in each P2RY_12_ mutant were colored as follows: white, original; slate, E188A; orange, E188A and R265A; magenta, R165A; pink, D266A; cyan, E188A and D266A; yellow, R265A and D266A; green, E188A and R265A and D266A. **(F)** A view of the P2RY12 mutant/Rg5 complex.

To find out the exact binding sites, we first carried out molecular docking analysis of Rg5 and P2RY_12_. As shown in **Figures 3A, B**, Rg5 was predicted to interact with P2RY_12_ at amino acid residues E188, R256, T260, R265, and D266. Furthermore, energy decomposition of amino acid residues based on MM/PBSA free energy calculation showed that amino acid residues S101, E188, R256, R265, and D266 were important in the binding process of Rg5 and P2RY_12_ (**Figure 3C**). Because R256 was reported to play a pivotal role for the binding of ADP with P2RY_12_, and the mutation of R256 weakened the activation of P2RY_12_ by ADP, we chose E188, R265, and D266 for site-directed mutagenesis. As shown in **Figure 3D**, a series of P2RY_12_ variants were generated and overexpressed in CHO cells followed by activation of 2MesADP. However, Rg5 still could antagonize 2MesADP-induced phosphorylation of AKT and ERK unless E188 and R265 was simultaneously mutated into alanine. According to the docking results in **Figure 3D**, there were two main binding modes of Rg5 in P2RY_12_ mutants. As shown in **Figure 3E**, Rg5 mainly bound to two extracellular pockets, which were separated by residues Y105 and K280. Pocket 2 was the binding site of Rg5 in the WT conformation, E188A mutant, and E188A and R265A mutant of P2RY_12_; whereas in other mutants, Rg5 bound to the more advantageous Pocket 1. The interaction between Rg5 and binding pocket in **Figure 3F** showed the absence of hydrogen bonding with E188 and R265 in the interaction of Rg5 and E188A and R265A mutant, which resulted in the weakened binding effect of Rg5 to P2RY_12_. Ranking results in **Table 2** also verified that the E188A and R265A mutant had the weakest affinity with Rg5, suggesting that E188 and R265 play an important role in the interaction between Rg5 and P2RY_12_.

**Table 2:** Ranking results of Rg5 binding to P2RY_12_ mutation variants.

Mutation	Rank
E188A	0.647
R265A	0.635
D266A	0.631
E188A and R265A	0.626
E188A and D266A	0.633
R265A and D266A	0.656
E188A and R265A andD266A	0.649

### Rg5 reduced thrombosis and inflammatory response in DVT model mice

**Figure 4:**
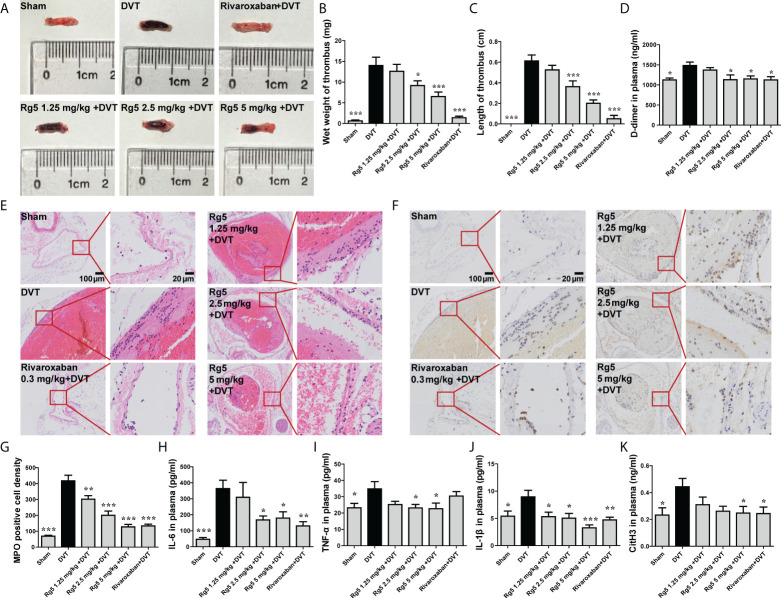
Effect of Rg5 administration on thrombosis and inflammation in DVT mice. **(A)** Morphology of venous thrombosis. **(B, C)** Thrombus wet weight and length (n = 8). **(D)** Plasma D-dimer content (n = 7). **(E)** HE staining of inferior vena cava. **(F)** MPO immunohistochemical staining of inferior vena cava. **(G)** MPO positive cell density (n = 6). **(H–K)** Plasma contents of IL-6, IL-1β, TNF-α, and CitH3 (n = 7). Data are shown as the mean ± SEM. *P < 0.05, **P < 0.01, ***P < 0.001 vs. DVT group.

As shown in **Figure 4**, IVC ligation caused significant thrombosis in mice (**Figure 4A**). Accordingly, the wet weight and length of thrombus in the DVT group mice were increased markedly, compared with the sham group (*P <* 0.001). Rg5 pretreatment, especially at dosages of 2.5 and 5 mg/kg, significantly reduced thrombus formation (*P <* 0.05 or *P <* 0.001). Meanwhile, Rg5 pretreatment at higher dosages suppressed the plasma D-dimer content in DVT mice (**Figure 4D**, *P <* 0.05). Similarly, the positive control drug, Rivaroxiban (0.3 mg/kg), also attenuated thrombus formation, as well as the plasma D-dimer content in DVT mice. These results demonstrated that Rg5 could prevent thrombosis in deep vein. On the other hand, DVT is closely relevant to thrombophlebitis. As shown in **Figure 4E**, much more inflammatory cells infiltrated in the veins of DVT mice compared with the sham mice. Moreover, MPO staining exposed that most of the accumulated inflammatory cells were neutrophils (**Figure 4F**). After pretreatment of Rg5, the infiltration of inflammatory cells including neutrophils in vein was reduced (*P <* 0.01 or *P <* 0.001). Rg5 treatment (2.5 and 5 mg/kg) also significantly inhibited the release of inflammatory cytokines IL-6, TNF-α, and IL-1β in plasma (**Figures 4H–J**, *P <* 0.05 or *P <* 0.001). In addition, DVT is closely related to NETs produced by neutrophils. As the biomarker of NET formation, the content of citrullinated histone H3 (CitH3) was elevated after induction of DVT, which was significantly reduced after Rg5 (5 mg/kg) pre-administration (**Figure 4K**, *P <* 0.05). Interestingly, the positive drug, Rivaroxiban (0.3 mg/kg), could also suppress neutrophil infiltration and inflammatory response, as well as CitH3 expression. These results suggested that Rg5 could inhibit NET release and inflammatory response in mice.

### Rg5 inhibited NETosis depending on P2RY_12_

**Figure 5:**
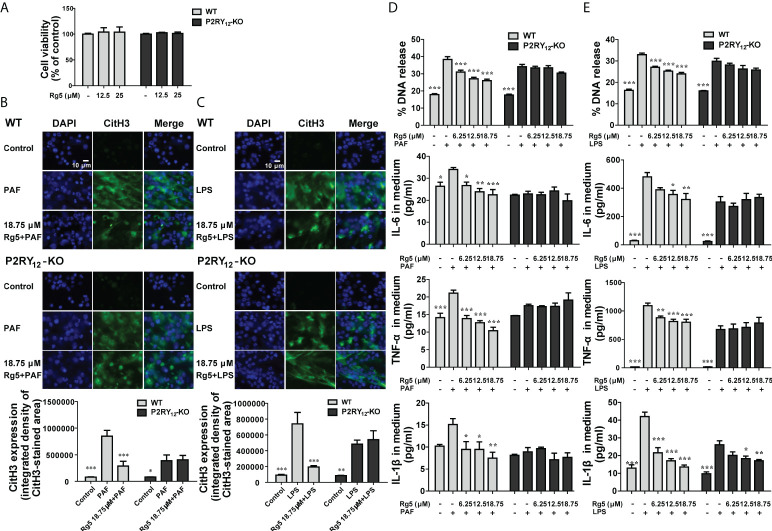
Effects of Rg5 on PAF or LPS-induced release of NETs and inflammatory factors in WT or P2RY_12_-KO neutrophils. **(A)** Effect of Rg5 on WT or P2RY_12_-KO neutrophils viability (n = 4). **(B, C)** Immunofluorescent staining and statistical analysis of CitH3 in WT or P2RY_12_-KO neutrophils stimulated by PAF or LPS. (n = 6). **(D, E)** DNA concentration (n = 4) and IL-6, IL-1β, and TNF-α concentrations (n = 4) in the medium of WT or P2RY_12_-KO neutrophils stimulated by PAF or LPS. All the data are shown as the mean ± SEM. *P < 0.05, **P < 0.01, ***P < 0.001 vs. PAF or LPS group.

On the premise that Rg5 had no effect on neutrophils viability (**Figure 5A**), we first investigated whether Rg5 had an inhibitory effect on NETosis induced by PAF or LPS. As shown in **Figure 5B**, Rg5 pre-treatment significantly reduced the chromatin depolymerization and the expression of CitH3 in PAF-induced WT neutrophils (*P <* 0.001). However, in PAF-induced P2RY_12_-KO neutrophils, Rg5 did not show the same effect. Similar results were found in LPS-induced WT and P2RY_12_-KO neutrophils (**Figure 5C**). In addition, Rg5 treatment dose-dependently reduced cfDNA release and inflammatory factor production in culture medium of WT neutrophils induced by PAF and LPS (**Figures 5D, E**). However, in PAF-induced P2RY_12_-KO neutrophils, Rg5 pre-treatment at 18.75 μM slightly suppressed the cfDNA release, whereas it could not inhibit further inflammatory factor release (**Figure 5D**). Similarly, in LPS-induced P2RY_12_-KO neutrophils, Rg5 still showed slight inhibitory effects on the production of IL-1β (**Figure 5E**). These results suggested that Rg5 inhibited NETosis mainly through P2RY_12_.

### Rg5 attenuated LPS or PAF-induced activation of PAD4 and ERK/NF-κB signaling pathway in neutrophils *via* P2RY_12_

**Figure 6:**
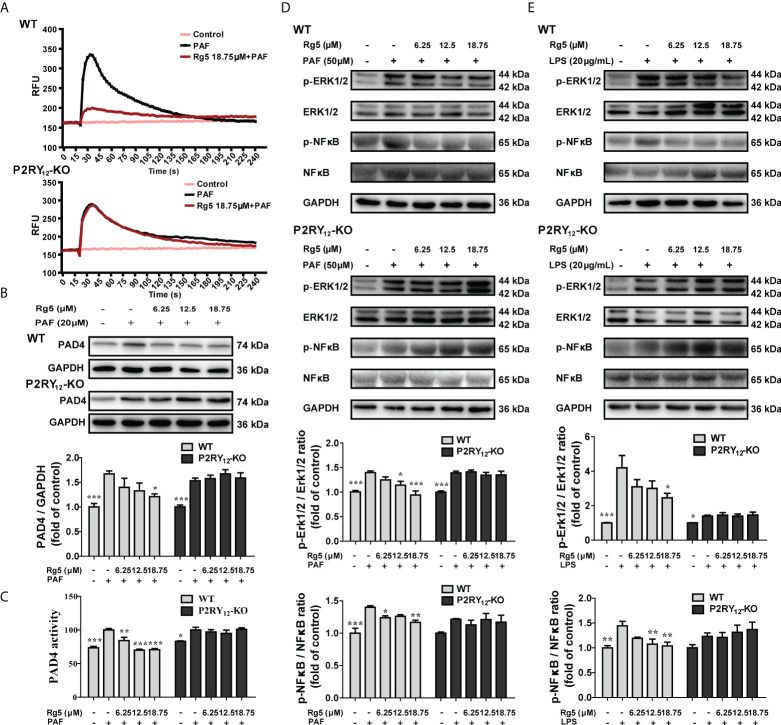
Effects of Rg5 on PAF or LPS-induced activation of PAD4 and ERK/NF-κB signaling pathway in WT or P2RY_12_-KO neutrophils. **(A)** Calcium flux in WT or P2RY_12_-KO neutrophils stimulated by PAF (n = 3). **(B)** Protein expression of PAD4 in WT or P2RY_12_-KO neutrophils stimulated by PAF (n = 5). **(C)** Activity of PAD4 in WT or P2RY_12_-KO neutrophils stimulated by PAF (n = 4). **(D, E)** Western Blot results and statistical analysis of protein expression and phosphorylation levels of ERK (n = 5) and NF-κB (n = 5) in WT or P2RY_12_-KO neutrophils stimulated by PAF or LPS. All the data are shown as the mean ± SEM. *P < 0.05, **P < 0.01, ***P < 0.001 vs. PAF or LPS group.

As PAD4 plays an important role in NETosis, we next examined whether Rg5 could influence the activity of PAD4 and whether this action was dependent on P2RY_12_. As shown in **Figure 6A**, Rg5 treatment significantly reduced PAF-induced calcium flux. In contrast, Rg5 could not further mitigate PAF-induced calcium flux in P2RY_12_-KO neutrophils (**Figure 6A**). In terms of PAD4 expression, 18.75 μM Rg5 treatment significantly mitigated the increase of PAD4 induced by PAF in WT neutrophils (**Figure 6B**, *P <* 0.05). Accordingly, the catalytic activity of PAD4 in WT neutrophils was suppressed by Rg5, compared with PAF group cells (**Figure 6C**, *P <* 0.01 or *P <* 0.001). When P2RY_12_ was deleted, Rg5 displayed no effect on the expression and activity of PAD4 in neutrophils. These results implicated that Rg5 suppressed PAF-induced elevation of PAD4 in neutrophils through P2RY_12_. Thereby, we investigated whether Rg5 could regulate the signaling pathway associated with inflammatory cytokine production and whether this effect was dependent on P2RY_12_. As shown in **Figure 6D**, PAF-induced a marked rise in the phosphorylation of ERK and NF-κB, which could be counteracted by Rg5 treatment (*P <* 0.05, *P <* 0.01, or *P <* 0.001). PAF could also induce the phosphorylation of ERK and NF-κB in P2RY_12_-KO neutrophils. However, Rg5 treatment did not suppress the activated ERK and NF-κB in P2RY_12_-KO cells. Similarly, Rg5 failed to inhibit LPS-activated ERK/NF-κB pathway in P2RY_12_-KO neutrophils (**Figure 6E**). These results indicated that Rg5 suppressed the inflammatory pathway in neutrophils depending on P2RY_12_.

## Discussion and conclusions

In this study, we demonstrated that Rg5 inhibited signal transduction in neutrophils and reduced DVT formation by interacting with P2RY_12_. On the one hand, Rg5 suppressed inflammatory response by inhibiting cytokine production through ERK/NF-κB signaling pathway; on the other hand, it reduced NETosis by preventing intracellular calcium mobilization, leading to the loss of PAD4 activity. Our results suggested that Rg5 might be a promising candidate for the prevention of DVT by counteracting neutrophil activation through P2RY_12_.

P2RY_12_ has been shown as a drug target for the prevention of platelet aggregation for a long time and is also involved in inflammation. For instance, knockout of P2RY_12_ or blocking of P2RY_12_ with Clopidogrel significantly reduced the volume of venous thrombosis in mice (18, 32). Clopidogrel, Cangrelor, and Ticagrelor have been used widely in clinic for the treatment of thrombogenesis by acting as P2RY_12_ antagonists, but some patients experienced side effects such as bleeding or recurrent ischemia (33). Interestingly, in our experiment, the injection of Rg5 could prolong the coagulation time compared with that of Cangrelor, but the bleeding time of Rg5 was shorter than that of Cangrelor (data not shown), suggesting that Rg5 might have less bleeding risk compared with Cangrelor. However, further research remains to be done in the future to confirm the effect.

Clopidogrel has been proven to inhibit the production of proinflammatory mediator in plasma, particularly IL-6, TNFα, and IL-1β, and reduce platelet–neutrophil interactions (18, 21, 34). However, whether P2RY_12_ plays an important role in the activation of neutrophils has not been clearly elucidated. A recent report demonstrated that, in the sepsis model of P2RY_12_-KO mice, there was no prominent increase of neutrophils in the serum and inflammation sites (35). However, there was no further investigation to disclose the underlying mechanism. In the present study, we demonstrated that both LPS and PAF stimulation enhanced inflammatory response and NET release in neutrophils; however, the effect of which was weakened in P2RY_12_-deficient neutrophils. These results implicated that P2RY_12_ is important for NETosis.

Rg5 has been shown to have multiple pharmacological activities, including anti-inflammatory, antitumor, neuroprotective, and cardioprotective properties (21). However, up to date, there is no report that clearly clarifies the direct molecular target. In our virtual screening, we found that Rg5 showed strong affinity with P2RY_12_, which was similar to Ticagrelor, a well-known P2RY_12_ antagonist. To confirm the binding, we conducted SPR assay and MD simulation, respectively, which revealed that Rg5 could dose-dependently bind to P2RY_12_ and form stable complex rapidly. Moreover, site-mutation analysis exposed that two amino acid residues E188 and R265, which did not directly interact with 2MesADP (36), were critical sites for the binding of Rg5 to P2RY_12_ and activation of downstream signaling. The roles of these two amino acids have been mentioned in previous studies, both of which were related to the activation of P2RY_12_ (37, 38). However, a study has also indicated that E188 and R265 were not the active sites for P2RY_12_; they mainly affect the conformational states of the protein (26). In agreement with the conjecture, the P2RY_12_ mutant constructed by homologous modeling suggested that the allosteric effect caused by mutations of E188 and R265 might account for the diminished antagonism of Rg5 to P2RY_12_. These results robustly corroborated the direct binding and antagonic activity of Rg5 to P2RY_12_.

Platelet aggregation and inflammation are related to the pathogenesis of DVT. During the occurrence of DVT in clinic, slow or restricted blood flow in veins leads to damage of venous endothelial cells due to hypoxia and further releases cytokines such as inflammatory factors, PAF, and chemokines, which induces the activation and aggregation of platelets and white blood cells. When systemic or local infection occurs, in addition to similar responses described above, platelets and leukocytes activate rapidly in response to direct pathogen stimulation, leading to a significantly increased risk of DVT (39). Activated platelets are recruited and transported to the venous wall where they attach directly to endothelial cells or to leukocytes to form heterogeneous aggregates, which is a critical step in DVT initiation (40). P2RY_12_ inhibitors Ticagrelor and Clopidogrel have been shown to significantly reduce the formation of platelet–neutrophil or platelet–monocyte aggregates and improve systemic inflammatory responses (41). The process of thrombosis is usually accompanied by the transformation of acute inflammation to chronic inflammation, and inflammation is closely related to DVT (42, 43). Recent studies have found that inflammation factors like IL-6, IL-1, and TNF-α were elevated in both DVT patients and mice (44, 45) and activated platelets and coagulation system, which further promoted thrombosis (46, 47). Neutrophils are essential in the development of thrombotic inflammation in DVT. In the DVT mouse model, 1 h after inferior vena cava stenosis, white blood cells began to appear and adhere to the venous endothelium. Six hours later, white blood cells covered the entire endothelial surface, of which more than 80% were neutrophils and the remaining 20% were monocytes. Moreover, when neutrophils are eliminated, DVT formation will be inhibited, indicating that the importance of neutrophils in DVT occurrence cannot be ignored (48). Activated neutrophils release NETs, which were found in the plasma and thrombus of patients with DVT (49, 50). Although monocytes also release extracellular traps (51), studies showed that neutrophils were the source of these extracellular traps (48), and a large number of NETs were also found in the thrombosis model (52). Meanwhile, treatment with DNase 1, known to degrade NETs, reduced the frequency of thrombosis (53), indicating that neutrophils and NETs in DVT are functionally important. In this study, we found that Rg5 inhibits DVT formation by reducing the release of inflammatory cytokines and the expression of NET biomarker CitH3 in mice. We induced bacterial and aseptic inflammation *in vitro* with LPS and PAF, respectively, and found that Rg5 reduced the inflammatory response of neutrophils by inhibiting P2RY_12_. This study focused only on the antagonistic effect of Rg5 against P2RY_12_ of neutrophils, rather than that of the platelets or other blood cells, like monocytes. However, Rg5 has been reported to have a significant anti-platelet aggregation effect (54). Combined with the fact that Rg5 can inhibit the phosphorylation of AKT and ERK signals downstream of P2RY_12_ and antagonize its activity, we speculate that Rg5 can inhibit platelet activation and platelet–white blood cell interaction by targeting P2RY_12_. Therefore, we believe that Rg5 may be an ideal treatment option for DVT.

P2RY_12_, as a member of the GPCR family, transmits signals mainly through two pathways: one modulates the cAMP level and PI3K/AKT pathway by binding to Gi protein, and the other influences Mitogen activated protein kinases (MAPK) signaling pathways such as ERK pathway by binding to β-arrestin or Gi protein (55). Previous studies have reported that the transcription and expression of inflammatory cytokines such as TNF-α, IL-1β, and IL-6 were related to the phosphorylation of NF-κB signal (56), which was affected by ERK pathway (57). PAD4 is highly expressed in neutrophil nuclei and relies on calcium mobilization to catalyze the conversion of several arginine sites on histones into citrulline (58), which plays an important role in the formation of NETs (59, 60). It has also been found that Rg5 can play an anti-inflammatory role by interfering NLRP3 signaling pathway and reducing inflammatory cytokines, whereas PAD4 also has the ability to upregulate NLRP3 inflammatory granules through post-transcription (61). Meanwhile, the activation of P2RY_12_ by ADP is closely associated with NLRP3- and NF-κB–mediated inflammatory responses (62). Therefore, on the basis of the inhibitory effect of Rg5 on P2RY_12_ activation in this study, the interference of Rg5 on NLRP3 signaling *via* P2RY_12_ may also explain the inhibitory effect of Rg5 on neutrophil inflammation. In the present study, Rg5 was found to inhibit ERK/NF-κB activation and inflammatory factor production in neutrophils induced by both LPS and PAF. Meanwhile, Rg5 restrained calcium influx, PAD4 activity, and NET release in PAF/LPS-induced neutrophils. However, in P2RY_12_ deficiency neutrophils, the suppressive effects of Rg5 on the aforementioned parameters were diminished. These results implicated that Rg5 modulated ERK/NF-κB and cAMP/PAD4 signaling pathway in neutrophils depending on P2RY_12_.

In summary, this study demonstrated that Rg5 as a natural P2RY_12_ antagonist could inhibit NETosis during thrombosis to slow down the formation of DVT. Our study provides a theoretical basis for the clinical application of Rg5 for the prevention of DVT.

## Data availability statement

The raw data supporting the conclusions of this article will be made available by the authors, without undue reservation.

## Ethics statement

The animal study was reviewed and approved by Shanghai University of Traditional Chinese Medicine.

## Author contribution

ZC, GW, and XX performed the experiments, analyzed the data, and wrote the paper. ZW and XW designed the study. HL, JL, and HS participated in its design and helped to draft the manuscript. MC and SL gave valuable suggestions about the study. All authors contributed to the article and approved the submitted version. 

## Funding

This work was supported by Shanghai Municipal Natural Science Foundation (22ZR1461100). Major International (Regional) Joint Research Project of NSFC (81920108033), Guangxi Science and Technology Base and Talent Special Project (Guike AD20297068).

## Conflict of interest

The authors declare that the research was conducted in the absence of any commercial or financial relationships that could be construed as a potential conflict of interest.

## Publisher’s note

All claims expressed in this article are solely those of the authors and do not necessarily represent those of their affiliated organizations, or those of the publisher, the editors and the reviewers. Any product that may be evaluated in this article, or claim that may be made by its manufacturer, is not guaranteed or endorsed by the publisher.
